# Longitudinal analysis of health care costs in patients with childhood onset inherited retinal dystrophies compared to healthy controls

**DOI:** 10.1186/s12886-022-02708-0

**Published:** 2022-12-02

**Authors:** Line Kessel, Jakob Kjellberg, Rikke Ibsen, Annette Rasmussen, Kamilla Rothe Nissen, Morten la Cour

**Affiliations:** 1 Department of Ophthalmology, Copenhagen University Hospital – Rigshospitalet-Glostrup, Valdemar Hansens Vej 1-23. 2600, Glostrup, Denmark; 2 Department of Clinical Medicine, University of Copenhagen, Copenhagen, Denmark; 3 VIVE, The Danish Center for Social Science Research, Copenhagen, Denmark

**Keywords:** Inherited retinal dystrophy, Childhood visual impairment, Health care costs. Injury

## Abstract

**Background:**

We evaluated health care costs in patients with childhood onset visual impairment caused by inherited retinal dystrophies (IRD).

**Methods:**

The IRD cohort, identified from the Danish Registry of Blind and Partially Sighted Children, was compared to age- and sex-matched controls from the national, Danish population registry. Information on health care expenditures for somatic and psychiatric in- and outpatient services, purchase of prescription medications and paid assistance at home were obtained from national registries for the years 2002–2017.

**Results:**

We included 412 in the IRD cohort (6,290 person years) and 1656 (25,088 person years) in the control cohort. Average, annual health care expenditures from age 0–48 years of age were €1,488 (SD 4,711) in the IRD cohort and €1,030 (4,639) in the control cohort. The largest difference was for out-patient eye care (13.26 times greater, 95% confidence interval 12.90–13.64). Psychiatric in-patient expenditures were 1.71 times greater (95% CI 1.66–1.76) in the IRD cohort but psychiatric out-patient health care costs were comparable between groups.

**Conclusions:**

Health care costs were approximately 40% greater in the IRD cohort compared to an age- and sex-matched sample from the general Danish population. This is relevant in the current situation with a number of trials aimed at treating IRDs using genetically based therapies. Although eye care expenditures were many times greater, they made up < 10% of the total health care expenditures even in the IRD cohort. The reduced costs related to injuries in the visually impaired cohort was a surprising finding but may reflect a reduced propensity to seek medical care rather than a reduced risk of injuries.

## Background

Childhood blindness is thought to affect 14 million children world-wide and whereas preventable causes dominate in developing nations, e.g. retinopathy of prematurity and cataract, inherited retinal dystrophies are a common cause of blindness and visual impairment in developed nations. [1] With the advent of expensive genetically based therapies such as voretigene neparvovec [2] there has been an increased focus on the health and economic benefits associated with treating inherited retinal diseases. However, no large scale direct evaluations are available. Previous reports have been based on indirect assumptions of expenditures [3, 4] or direct evaluation of a few affected patients, e.g. via questionnaires, which have been extrapolated to larger cohorts. [5, 6].

A substantial proportion of those affected by childhood onset retinal dystrophies have extra-ocular disease, e.g. Usher disease, Bardet-Biedl, or neuronal ceramide lipofuscinosis. [7, 8] Even those with non-syndromic childhood onset retinal dystrophy may require extra medical attention to evaluate or monitor general growth and development. Missing an important sensory function, such as vision, may influence early development, e.g. autistic features have been described among congenitally blind children [9] and mental and behavioral disorders are common in children with Usher syndrome. [10] Physical disease may also be more prevalent among those with visual impairment, e.g. visual impairment in adults is known be associated with an increased risk of falls [11] which may increase health care costs related to the management of injuries. Severe disease with onset in childhood may have repercussions extending well into adult life, e.g. survivors of childhood cancer report reduced quality of life especially for physical well-being compared to their siblings. [12].

The aim of the study was to assess health care costs in a cohort of patients with childhood onset retinal dystrophies without severe systemic comorbidities and to compare to an age- and sex-matched cohort drawn from the background population. We used comprehensive, national Danish registries to obtain a complete picture of somatic and psychiatric health care costs in both primary and secondary health care settings.

## Methods

We compared health care expenditures in a cohort of individuals with childhood-onset inherited retinal dystrophies (IRD) without severe systemic comorbidities to an age- and sex-matched sample from the background population.

### Childhood-onset inherited retinal dystrophy (IRD) cohort

The IRD cohort was identified from The Danish Registry for the Blind and Partially Sighted Children which is a national registry of all children (< 18 years) with visual impairment or blindness defined as visual acuity ≤ 6/18, hemianopia or visual field < 20 degrees on the better seeing eye. In addition, all children with progressive retinal disease must be registered at the time of diagnosis irrespective of visual function. The registry covers the years since 1970 and includes 609 patients with childhood onset inherited retinal disease. For the present study, we included patients with visual impairment from IRD but without severe comorbidities such as delayed psychomotor development, mental disability or severe somatic disease (*n* = 55) and those with insufficient quality of the medical records to determine whether the patient had severe systemic comorbidities (*n* = 39) leaving 515 patients with childhood-onset IRD eligible for the study.

### Control cohort

A control group was sampled 1:4 by matching the cases to controls from the Danish central person registry (CPR) by age, gender and index year. As data from the CPR were first available from 1980, the index year was the same as the year of registration in Registry for Blind and Partially Sighted Children for all registered after 1979 and for those registered before 1980, the index year and index age was the first year the case has a registration in the CPR after 1979. The control cohort was chosen to represent the general Danish population and was not controlled for severe comorbidities or other demographic variables than those described above.

### Health care expenditures

The average yearly health care expenditures per person were calculated based on national registries and were calculated for the entire available years (0–48 years of age) and further sub-grouped in age groups: 0–10, 11–20, 21–30 and 31–48 years of age. The average cost for each age group was calculated for the entire study period 2002–2017. All the years within the study period a subject was in a particular age group, the subject contributed with information to that particular age group, but a subject could change age group and be in different age groups during the study period.

Health care expenditures were broken down to costs related to:

- Primary healthcare sector, e.g. general practitioners, ophthalmologists, psychiatrist and psychologists working in private practice
- Secondary healthcare sector (hospitals) which was further broken down into somatic and psychiatric services and to out-patient services and in-patient admissions. Somatic in- and outpatient services were further subdivided into costs related to eye care (ICD10 diagnostic codes H00-H59), injuries (ICD10 diagnostic codes S00-T98) and all other (all ICD10 diagnostic codes not mentioned above)
- Prescription medication which was further sub-grouped into costs related to neurologic and psychiatric diseases, i.e. ATC groups N05 including all subgroups (antipsychotics, anxiolytics, hypnotics and sedatives) and N06 including all subgroups (antidepressants, drugs against ADHD and narcoleptics and dementia) and all other types of prescription medications (all ATC groups not mentioned above)
- Home care which was further divided into personal care and practical help

The calculated healthcare costs are based on data for the period 2002–20017 except for psychiatric secondary healthcare sector costs which were only available from 2004 and home care cost which was only available from 2009.

Data were linked between registries using the CPR number which is a unique code assigned to each Danish resident that is used for every interaction between an individual and public or private services such as health care, taxation, education etc. We accessed the following registries for the study:

- The socio-demographic register (BEF), available from 1980-2019
- Health care registers for secondary health care services was available from 2002-2017 and included the Danish somatic patient registry (LPR), Danish psychiatric patient registry (LPR-PSYK, only available 2004-2017). We looked at costs related to in-patient services (DRG) and out-patient services (DAGS and BES), and sub-grouped as described above depending on diagnostic codes (DIAG). Furthermore, we accessed information on operations and other surgical or diagnostic procedures (OPR, SKSOPR and SKSUBE)
- Health care services for primary health care services were accessed via the Danish Health Insurance Register (Sygesikringsregisteret)
- Costs related to prescription medication were accessed via the Danish Drug Register (LMDB) and sub-grouped into therapeutic groups as described above
- Costs related to assistance provided in non-hospital settings (private homes or care homes, collectively termed “home care”) was sub-grouped into assistance with personal care or practical help and were available from 2009-2017 via the following variables: AEFV (practical assistance and personal care in private homes), AEPB (practical assistance and personal care in care homes), HJSP (nursing assistance at home), AETR (rehabilitation)

### Statistical methods

Actual costs are reported as average values with standard deviations in Euro per person per year. To test for differences between costs in the IRD and control cohort we used a 2-step one model generalized linear regression model (GLM) with a gamma distribution and link = log while we controlled for parental educational level. The 2-step one model regression was used since we estimated cost as continuous variables where some subjects had no expenditure (a “0” value in the response variable). An ordinary gamma model only includes positive values, but the 2-step can model data with 0’s. [13].

## Results

Health care expenditure information from the national, Danish registries covering the years 2002 to 2017 was available in 412 patients with childhood-onset inherited retinal disease (IRD) and 1656 unique age- and sex-matched control subjects corresponding to a total of 6,290 person years in the IRD group and 25,088 person years in the control group. Overall, health care costs were higher in the IRD cohort with an annual average of €1,488 (mean, standard deviation (SD) 4,711) compared to €1,030 (4,639) in the control cohort between the ages of 0 to 48 years (youngest and oldest observation during the study period), see Table 1.

**Table 1:** Average yearly healthcare costs in Euro per person from 0–48 years of age

	**IRD cohort** **Euro (mean (SD))**	**Control cohort** **Euro (mean (SD))**	**GLM model** **Estimate (95% CI)**	***P*-value**
Person years (n)	6290	25,088		
*Somatic health care costs*				
Outpatient Services	470 (1,202)	303 (1,496)	1.52 (1.48–1.56)	0.000
Eye	100 (349)	8 (153)	13.26 (12.90–13.64)	0.000
Injury	31 (134)	43 (241)	0.69 (0.67–0.71)	0.000
All other	339 (1,118)	252 (1,461)	1.32 (1.28–1.35)	0.000
Inpatient Admissions	415 (2,733)	309 (3,348)	1.33 (1.30–1.37)	0.000
Eye	13 (227)	2 (107)	20.04 (19.10–21.02)	0.000
Injury	32 (434)	37 (483)	0.84 (0.82–0.87)	0.000
All other	371 (2,622)	270 (3,284)	1.36 (1.33–1.40)	0.000
Prescription medication	146 (619)	93 (439)	1.41 (1.37)	0.000
ATC N05 and N06	42 (334)	26 (344)	1.22 (1.19–1.26)	0.000
All other	104 (507)	67 (255)	1.47 (1.43–1.51)	0.000
Primary health sector	236 (412)	177 (297)	1.31 (1.27–1.35)	0.000
Psychiatrist/psychologist	14 (112)	7 (76)	1.79 (1.74–1.84)	0.000
All other primary sector	222 (388)	170 (281)	1.29 (1.25–1.33)	0.000
*Total, somatic costs*	*1,267 (3,668)*	*882 (3,941)*	*1.40 (1.36–1.44)*	*0.000*
*Psychiatric health care costs*				
Psychiatric outpatient services	64 (575)	61 (314)	0.99 (0.96–1.02)	0.523
Psychiatric inpatient admissions	132 (2,519)	62 (1,817)	1.71 (1.66–1.76)	0.000
***Total costs, somatic and psychiatric***	***1,488 (4,711)***	***1,030 (4,639)***	***1.39 (1.35–1.43)***	***0.000***
*Home care*				
Home care—care	98 (1,335)	23 (954)	3.78 (3.64–3.92)	0.000
Home care—practical help	92 (566)	3 (106)	34.95 (33.63–36.25)	0.000

Costs are reported in Euro (mean (SD). The generalized linear regression model (GLM) was controlled for parental education

*IRD* inherited retinal dystrophy, *GLM* generalized linear regression model, *SD* standard deviation

Health care cost information broken down into a pediatric age group (0–10 years), youngsters (11–20 years), young adults (21–30) and adults (31–48 years) are available in Tables 2,3,4,5.

**Table 2:** Average yearly healthcare costs in Euro per person aged 0–10 years

	**IRD cohort** **Euro (mean (SD))**	**Control cohort** **Euro (mean (SD))**	**GLM model** **Estimate (95% CI)**	***P*-value**
Person years (n)	1402	5704		
*Somatic health care costs*				
Outpatient Services	406 (882)	149 (498)	2.69 (2.54–2.86)	0.000
Eye	126 (346)	7 (100)	17.74 (16.73–18.81)	0.000
Injury	31 (118)	29 (138)	1.10 (1.04–1.17)	0.002
All other	249 (781)	113 (452)	2.19 (2.06–2.32)	0.000
Inpatient Admissions	459 (3.655)	324 (2,274)	1.39 (1.31–1.47)	0.000
Eye	16 (189)	0 (19)	58.92 (54.26–63.98)	0.000
Injury	17 (273)	37 (506)	0.43 (0.40–0.46)	0.000
All other	426 (3.637)	287 (2,212)	1.46 (1.38–1.55)	0.000
Prescription medication	48 (211)	46 (208)	1.07 (1.01–1.13)	0.033
ATC N05 and N06	1 (22)	8 (109)	0.15 (0.14–0.16)	0.000
All other	47 (210)	38 (174)	1.26 (1.19–1.34)	0.000
Primary health sector	217 (321)	157 (235)	1.37 (1.29–1.45)	0.000
Psychiatrist/psychologist	0 (5)	0 (7)	1.04 (0.96–1.13)	0.325
All other primary sector	217 (321)	157 (238)	1.37 (1.29–1.45)	0.000
*Total, somatic costs*	*1,130 (4,169)*	*676 (2,527)*	*1.63 (1.54–1.73)*	*0.000*
*Psychiatric health care costs*				
Psychiatric outpatient services	7 (97)	19 (306)	0.37 (0.34–0.39)	0.000
Psychiatric inpatient admissions	-	13 (623)	-	-
***Total costs, somatic and psychiatric***	***1,145 (4,510)***	***687 (2,742)***	***1.62 (1.52–1.73)***	***0.000***
*Home care*				
Home care—care	-	-	-	-
Home care—practical help	-	-	-	-

Costs are reported in Euro (mean (SD). The generalized linear regression model (GLM) was controlled for parental education.—too few observations to tabulate data. IRD: inherited retinal dystrophy. GLM: generalized linear regression model. SD: standard deviation

**Table 3:** Average yearly healthcare costs in Euro per person aged 11–20 years

	**IRD cohort** **Euro (mean (SD))**	**Control cohort** **Euro (mean (SD))**	**GLM model** **Estimate (95% CI)**	***P*-value**
Person years (n)	1980	8048		
*Somatic health care costs*				
Outpatient Services	497 (1,270)	197 (780)	2.49 (2.37–2.61)	0.000
Eye	129 (404)	4 (0)	35.51 (33.80–37.30)	0.000
Injury	37 (158)	49 (255)	0.74 (0.70–0.78)	0.000
All other	331 (1,146)	144 (727)	2.26 (2.15–2.37)	0.000
Inpatient Admissions	360 (2,410)	182 (4,720)	2.03 (1.93–2.13)	0.000
Eye	17 (297)	1 (37)	54.24 (50.22–58.60)	0.000
Injury	46 (552)	27 (213)	1.74 (1.65–1.83)	0.000
All other	297 (2,234)	154 (4,709)	1.98 (1.88–2.08)	0.000
Prescription medication	94 (332)	84 (344)	1.07 (1.02–1.13)	0.008
ATC N05 and N06	33 (237)	33 (284)	0.81 (0.77–0.85)	0.000
All other	61 (228)	51 (165)	1.18 (1.12–1.24)	0.000
Primary health sector	178 (373)	130 (257)	1.34 (1.28–1.41)	0.000
Psychiatrist/psychologist	4 (48)	3 (45)	1.33 (1.26–1.39)	0.000
All other primary sector	173 (367)	127 (249)	1.34 (1.28–1.41)	0.000
*Total, somatic costs*	*1,129 (3,397)*	*593 (4,891)*	*1.90 (1.80–1.99)*	*0.000*
*Psychiatric health care costs*				
Psychiatric outpatient services	86 (769)	58 (555)	1.48 (1.41–1.56)	0.000
Psychiatric inpatient admissions	142 (3,461)	52 (1,939)	4.24 (4.02–4.47)	0.000
***Total costs, somatic and psychiatric***	***1,409 (5,130)***	***716 (5,570)***	***1.95 (1.85–2.05)***	***0.000***
*Home care*				
Home care—care	-	-	-	-
Home care—practical help	1 (26)	3 (141)	0.43 (0.39–0.47)	0.000

Costs are reported in Euro (mean (SD). The generalized linear regression model (GLM) was controlled for parental education.—too few observations to tabulate data

*IRD* inherited retinal dystrophy, *GLM* generalized linear regression model, *SD* standard deviation

**Table 4:** Average yearly healthcare expenditures per person in Euro from 21–30 years of age

	**IRD cohort** **Euro (mean (SD))**	**Control cohort** **Euro (mean (SD))**	**GLM model** **Estimate (95% CI)**	***P*-value**
Person years (n)	1619	6319		
*Somatic health care costs*				
Outpatient Services	452 (1,280)	372 (1,791)	1.21 (1.14–1.27)	0.000
Eye	60 (269)	11 (209)	18.99 (17.16–20.97)	0.000
Injury	29 (131)	51 (257)	0.56 (0.53–0.59)	0.000
All other	364 (1,224)	311 (1,744)	1.16 (1.09–1.22)	0.000
Inpatient Admissions	432 (2,637)	346 (1,993)	1.23 (1.17–1.30)	0.000
Eye	4 (120)	0 (19)	60.24 (54.53–66.54)	0.000
Injury	35 (493)	54 (634)	0.61 (0.58–0.64)	0.000
All other	394 (2,427)	292 (1,754)	1.33 (1.26–1.40)	0.000
Prescription medication	152 (553)	115 (616)	1.26 (1.19–1.33)	0.000
ATC N05 and N06	63 (459)	35 (552)	2.35 (2.22–2.48)	0.000
All other	89 (289)	80 (264)	1.10 (1.04–1.16)	0.001
Primary health sector	250 (357)	206 (275)	1.21 (1.14–1.27)	0.000
Psychiatrist/psychologist	27 (158)	14 (105)	1.90 (1.80–2.01)	0.000
All other primary sector	224 (300)	192 (244)	1.16 (1.10–1.22)	0.000
*Total, somatic costs*	*1,287 (3,626)*	*1,039 (3,157)*	*1.22 (1.15–1.28)*	*0.000*
*Psychiatric health care costs*				
Psychiatric outpatient services	70 (513)	92 (751)	0.67 (0.63–0.71)	0.000
Psychiatric inpatient admissions	178 (2,257)	114 (2,482)	1.05 (0.99–1.12)	0.095
***Total costs, somatic and psychiatric***	***1,520 (4,575)***	***1,283 (4,327)***	***1.13 (1.07–1.20)***	***0.000***
*Home care*				
Home care—care	-	-	-	-
Home care—practical help	79 (466)	3 (74)	82.96 (73.53–93.59)	0.000

Costs are reported in Euro (mean (SD). The generalized linear regression model (GLM) was controlled for parental education.—too few observations to tabulate data

*IRD* inherited retinal dystrophy, *GLM* generalized linear regression model, *SD* standard deviation

**Table 5:** Average yearly health care expenditures in Euros per person aged 31–48 years of age

	**IRD cohort** **Euro (mean (SD))**	**Control cohort** **Euro (mean (SD))**	**GLM model** **Estimate (95% CI)**	***P*-value**
Person years (n)	1289	5017		
*Somatic health care costs*				
Outpatient Services	518 (1,289)	561 (2,402)	0.91 (0.86–0.97)	0.003
Eye	77 (343)	11 (206)	7.31 (6.85–7.80)	0.000
Injury	23 (115)	41 (286)	0.52 (0.49–0.55)	0.000
All other	419 (1,232)	509 (2,377)	0.82 (0.77–0.87)	0.000
Inpatient Admissions	433 (2,065)	448 (3,052)	1.00 (0.94–1.06)	0.961
Eye	14 (251)	6 (232)	6.34 (4.85–8.29)	0.000
Injury	24 (252)	33 (460)	0.78 (0.73–0.83)	0.000
All other	395 (2,033)	410 (2,984)	1.00 (0.94–1.07)	0.905
Prescription medication	324 (1,107)	134 (493)	2.17 (2.04–2.31)	0.000
ATC N05 and N06	73 (435)	25 (256)	1.91 (1.79–2.03)	0.000
All other	251 (997)	109 (396)	2.19 (2.06–2.33)	0.000
Primary health sector	327 (572)	238 (408)	1.36 (1.28–1.45)	0.000
Psychiatrist/psychologist	26 (161)	14 (108)	1.78 (1.67–1.89)	0.000
All other primary sector	301 (540)	224 (388)	1.33 (1.25–1.42)	0.000
*Total, somatic costs*	*1,602 (3,519)*	*1,381 (4,360)*	*1.13 (1.07–1.20)*	*0.000*
*Psychiatric health care costs*				
Psychiatric outpatient services	78 (565)	71 (725)	0.98 (0.92–1.05)	0.577
Psychiatric inpatient admissions	185 (2,318)	65 (1,444)	0.99 (0.85–1.15)	0.863
***Total costs, somatic and psychiatric***	***1,867 (4,390)***	***1,525 (4,790)***	***1.16 (1.09–1.24)***	***0.000***
*Home care*				
Home care—care	-	-	-	-
Home care—practical help	249 (944)	4 (104)	158.33 (147.61–169.83)	0.000

Costs are reported in Euro (mean (SD). The generalized linear regression model (GLM) was controlled for parental education.—too few observations to tabulate data

*IRD* inherited retinal dystrophy, *GLM* generalized linear regression model, *SD* standard deviation

The larger expenditure in the IRD cohort was in part explained by higher costs related to both in- and out-patient eye-related health care but the costs for all types of somatic health care were higher in the IRD cohort except for injuries where the costs were lower in the IRD cohort. There was no significant difference in costs related to psychiatric out-patient disease, but costs associated with psychiatric inpatient admissions were higher in the IRD cohort (€132 versus €62 in the control cohort). Costs related to mental health (including prescription medication, psychiatrists, psychologists and in- and outpatient psychiatric services) went from < 6% in the youngest age group to 11–20% in the other age groups.

**Fig. 1:**
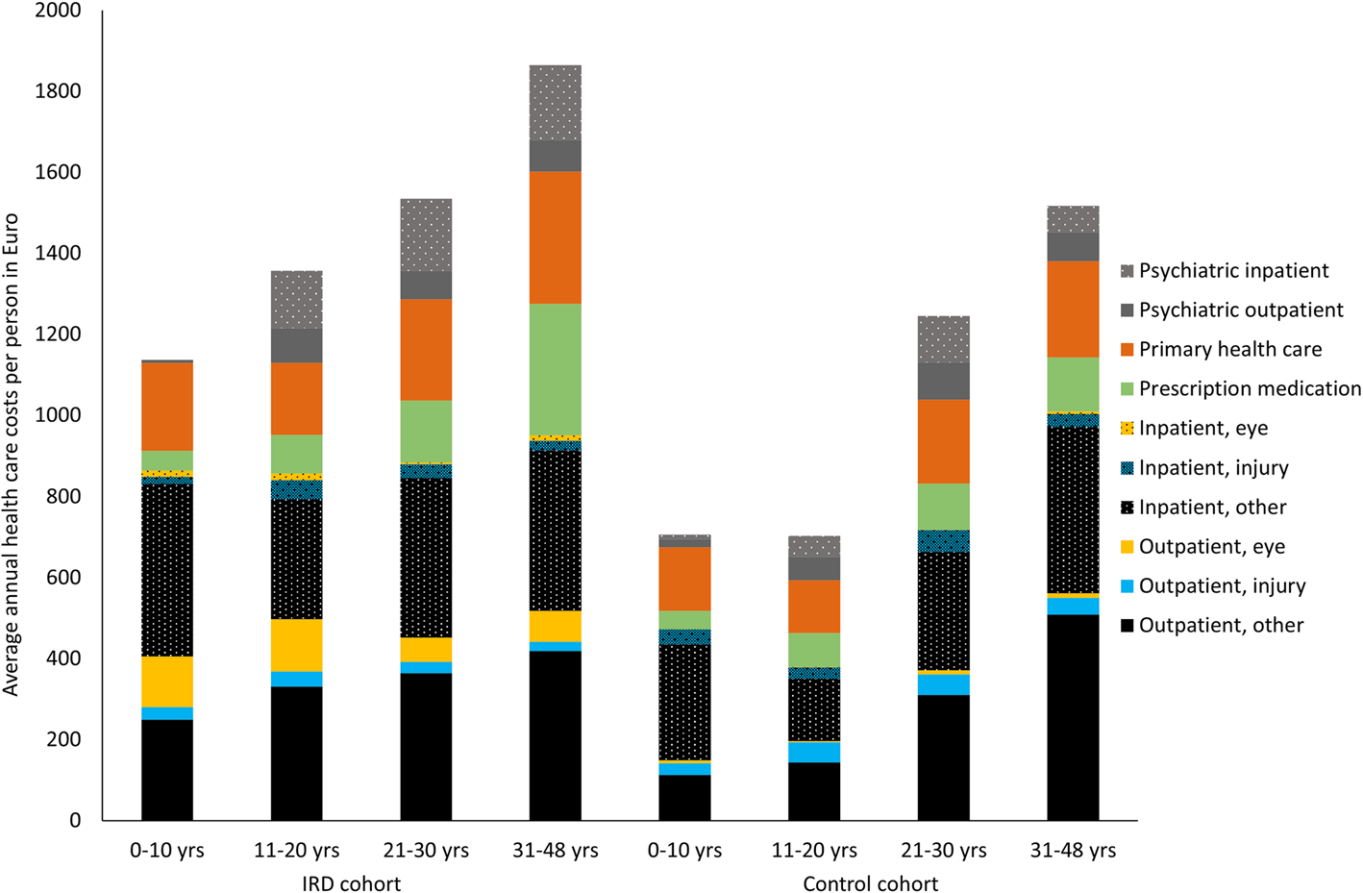
Graphic presentation of the average annual health care costs per decade for the IRD and control cohorts

Average, annual health care costs increased with age in both the IRD and control cohorts, but the increase was larger in the control cohort from €687 at age 0–10 to €1,525 at 30–48 years of age versus €1,145 to €1,867 in the IRD cohort, see Fig. 1 and Tables 2,3,4,5. In the pediatric population (0–10 years) costs related to inpatient admissions, particularly those that were not related to eye care or injuries, constituted the largest proportion of health care expenditures but after 10 years of age, outpatient services became the most costly health care item. The primary sector took up around 16% of the health care costs with small variations over the age groups and between the IRD and control cohort but the as the total costs was higher in the IRD group, the actual costs of primary care was also higher with an average annual expenditure of €236 versus €177 in the control group for the entire study period.

The proportion of health care costs related to eye care (inpatient admissions and outpatient services) reduced with age from 12% of all health care costs in the pediatric IRD cohort (Table 2) to 10% in the 11–20 year old (Table 3) and stabilized at 4–5% after 21 years of age (Tables 4 and 5). Costs related to eye care constituted around or less than 1% of all costs in the control cohort at all ages.

Costs related to practical assistance at home was negligible in the control cohort at all ages and in the IRD cohort before 21 years of age. At 21–30 years of age, the average annual costs related to practical assistance at home was €79 and at 31–48 years of age it was €249 in the IRD cohort.

## Discussion

We evaluated health care costs in a cohort of patients who had childhood onset inherited retinal dystrophies (IRD) and who had been registered in the nation-wide, Danish Registry for Blind and Partially Sighted Children. The registry includes children < 18 years of age with a visual impairment defined as visual acuity ≤ 20/60 or significant visual field defects (i.e. < 20 degrees or hemianopia) or a progressive retinal dystrophy. It is mandatory for doctors to ensure registration of relevant children. Thus, we expect our IRD cohort to be a complete sample of Danish childhood onset IRD patients. We used comprehensive national registries to evaluate health care costs and were able to include all costs both from the primary and secondary health care sector. Health care in Denmark is largely tax-financed without significant contributions from private insurance companies. Especially for health care related to IRD, there is no private system. This means that all Danish residents will have access to the same health care irrespective of income or insurance and that all health care expenditures were available from the national registries. Children with inherited retinal dystrophies have a high prevalence of systemic comorbidities [7] which may contribute to the overall need for health care. We only included patients without severe comorbidities as we wanted to evaluate the effect of the visual impairment itself rather than the syndromes or systemic diseases. Thus, health care costs in those with childhood-onset IRD with syndromic manifestations are expected to be larger than what we found.

Not surprisingly, we found that the IRD cohort had higher health care expenditures for eye-related health care services since childhood both for in- and outpatient eye services when compared to a sex- and age-matched cohort drawn from the background population. Overall, health care costs were significantly higher in the IRD cohort than in the background population which was surprising as the IRD cohort had been selected to exclude those with severe somatic comorbidities related to the genetic defect, e.g. a person with Bardet-Biedl syndrome was included in the cohort when the medical files stated that the person was well-functioning and attended normal school but not when the medical file stated that the person had autistic features and attended special school.

Costs associated with practical assistance at home was negligible in childhood and adolescence in both the IRD and control groups but increased in the IRD group as they became adults. This most likely reflects that they chose to live independently of family members as adults. Although the costs related to practical assistance in the home was nearly 83 times greater in the IRD cohort, the benefits in terms of actual expenditures were small with a mean of €92 which corresponds to less than 6 h of paid assistance per person per year. The standard deviation of the estimate was, however, high (€566) which suggests that a few individuals from the IRD cohort received more help and many likely received no paid practical help at home. Presumably, costs associated with practical assistance could increase in the IRD cohort as they age.

It was surprising to find that health care costs related to injuries were lower in the IRD group than in the control group. Visual impairment is known be associated with an increased risk of falls [11] and reversing the visual impairment, e.g. by cataract surgery [14], reduces the risk of falls. However, the majority of studies related to injuries and visual impairment are based on older patients who may have many comorbidities [15]. Visually impaired children are often described by parents as clumsy and IRD patients themselves frequently describe difficulties navigating, bumping into or tripping over things [16]. One might speculate that the IRD cohort did not experience fewer injuries than the control cohort but that they were less likely to seek medical care as they and their families were used to deal with injuries from early childhood and would not seek medical assistance unless the injury was very severe. Another likely explanation could be that they did in fact experience less injuries because they were less physically active, e.g. reduced participation in after-school sport activities, or less likely to participate in high risk sport activities such as contact sport but our dataset did not allow us to test this hypothesis.

As a physician managing patients with IRD, it was very surprising to see that the costs associated with psychiatric and mental disease was the same in the IRD and control cohort. Our model included both costs related to in- and outpatient hospital settings, psychiatrists and psychologists working in the primary care sector and costs related to prescription medications. Mental issues are often mentioned by patients as one of the main obstacles in life, e.g. fear of becoming blind, feeling of being inadequate and inferior in society [16–19]. Dealing with mental health problems has been described by some as a major part of living with early-onset retinal dystrophies, such as x-linked retinitis pigmentosa [20], whereas others have not found an increase in psychiatric disease in patients with IRD [21]. It should, however, be noted that mental health problems do not equate psychiatric disease. While we did not find statistical differences in health care expenditures related to mental health, costs related to mental issues made up a substantial proportion (15–20%) of the total health care costs in both the IRD and control cohort in our relatively young population (oldest observation at 48 years of age).

## Conclusion

We found that health care costs in patients with childhood onset inherited retinal dystrophies were on average ~ 40% higher than in an age- and sex-matched background population and that this was only to some extent explained by larger costs related to eye care. This information is relevant for health authorities when evaluating the cost–benefit of new, often genetically based, therapies addressing IRDs.

## Data Availability

The data that support the findings of this study are available from Statistics Denmark (project number 707892) but restrictions apply to the availability of these data, which were used under license for the current study, and so are not publicly available. Data are however available from the authors upon reasonable request and with permission of appropriate legal bodies.
